# Detection of *bla*_NDM-1_ in *Stenotrophomonas maltophilia* isolated from Brazilian soil

**DOI:** 10.1590/0074-02760170558

**Published:** 2018-05-14

**Authors:** João Pedro Rueda Furlan, André Pitondo-Silva, Eliana Guedes Stehling

**Affiliations:** 1 Universidade de São Paulo, Faculdade de Ciências Farmacêuticas de Ribeirão Preto, Departamento de Análises Clínicas, Toxicológicas e Bromatológicas, Ribeirão Preto, SP, Brasil

**Keywords:** *bla*_NDM-1_, soil, Stenotrophomonas maltophilia, IncA/C

## Abstract

This study reports the presence of the *bla*_NDM-1_ gene in an isolate of *Stenotrophomonas maltophilia* obtained from a Brazilian soil, inside an IncA/C plasmid with ~ 45 Kb. To the best of our knowledge, this is the second report in the world and the first in Brazil of NDM-producing bacterium isolated from soil.

The main mechanism of resistance to β-lactams is the production of β-lactamases, which hydrolyzes these antibiotics with consequent loss of the antimicrobial effect against bacteria. Bush and Jacoby (2010) classified the β-lactamases into three functional groups and New Delhi Metallo-β-Lactamase (NDM) is one of the most important and worrying of group three, due to its wide spectrum of action on β-lactam antibiotics, except for aztreonam. NDM has been extensively researched and, although it is mostly described in clinical isolates, it has also been reported in bacteria isolated from soil and water (Wang and Sun 2015, Mahon et al. 2017).

The great majority of NDM-producing bacteria belongs to the *Enterobacteriaceae* family and nonfermenting Gram-negative bacilli (NFGNB), including *Acinetobacter baumannii* (Wang and Sun 2015, Mahon et al. 2017). In some locations in India and China, NDM is considered endemic. In other countries such as Colombia, Egypt and Saudi Arabia there are reports of outbreaks and some cases are described worldwide, including Brazil (Dortet et al. 2014).

Due to the high levels of antimicrobial resistance, research on the mechanisms of acquisition and transfer of resistance by different species of bacteria has been increasingly widespread; however, the great majority of studies has been carried out with clinical bacteria. In the meantime, the soil microbiota is considered a great reservoir of antimicrobial resistance genes with clinical relevance, which can disseminate to other sources, such as some β-lactamases encoding genes (Humeniuk et al. 2002, Wright 2007). Although the origin of Metallo-β-lactamases (MBLs) is unknown, a recent study showed the characterisation of new MBLs in bacteria isolated from soil (Gudeta et al. 2015). For this reason, this study aimed to investigate in bacteria isolated from soil the presence of the *bla*_NDM_ gene, which encodes an important β-lactamase little described in soil samples.

One hundred and fifty bacterial isolates from soil samples from different plantation areas in the five Brazilian regions was obtained according to Martins et al. (2014), using MacConkey Agar (Oxoid, United Kingdom) for to select Gram-negative bacteria. Genomic DNA was extracted using the QIAamp DNA Mini Kit (QIAGEN) according to the manufacturer's instructions and the bacterial identification was performed by polymerase chain reaction (PCR) followed by DNA sequencing of the 16S and 23S rRNA genes (Weisburg et al. 1991, Hunt et al. 2006). The amplicons were purified using the Illustra™ GFX™ PCR DNA Kit (GE Healthcare, USA) and submitted to DNA sequencing on an ABI 3500xL Genetic Analyzer platform (Applied Biosystems, USA). The obtained nucleotide sequences were compared with those available in GenBank using the BLAST algorithm (http://blast.ncbi.nlm.nih.gov/Blast.cgi).

Antimicrobial susceptibility testing was performed by the disc diffusion method, and the minimum inhibitory concentration (MIC) was determined by the broth dilution method, according to the Clinical and Laboratory Standards Institute (CLSI 2015). The genes *bla*_CTX-M_ - (groups 1, 2, 8, and 9), *bla*_CMY_, *bla*_KPC_, *bla*_GES_, *bla*_SHV_, *bla*_PER_, *bla*_VEB_, *bla*_OXA-48-_ *_like_*, *bla*_OXA-1-_ *_like_*, *bla*_IMP_, *bla*_VIM_, and *bla*_NDM_ were investigated by PCR using the primers and conditions described by Dallenne et al. (2010) and Peirano et al. (2011). The amplicon was purified, sequenced, and aligned using Clustal Omega EMBLEBI Multiple Sequence Alignment (http://www.ebi.ac.uk/Tools/msa/clustalo/).

Plasmid DNA was extracted using the Plasmid Midi Kit (QIAGEN) and purified after excision of the gel band with the QIAquick Gel Extraction Kit (QIAGEN), according to the manufacturer's instructions. Plasmids were screened by PCR-based replicon typing (Carattoli et al. 2005). The molecular weight of the plasmid found in the study was determined by comparison with standard plasmids of the reference strains *Escherichia coli* V517 (Macrina et al. 1978) and *E. coli* 39R861 (Pitondo-Silva et al. 2014) using BioNumerics software 5.1 (Applied Maths, Belgium), after standard 0.8 % agarose gel electrophoresis.

Among the analysed isolates, only one *Stenotrophomonas maltophilia* named S431 (GenBank accession number MF079262 and KY242787) was the only one that presented the *bla*_NDM-1_ gene (GenBank accession number MF589973). In addition, for this isolate, several other β-lactamases encoding genes above cited were researched and none of them was found. The *bla*_NDM-1_ gene was detected inside an IncA/C plasmid (*rep*A gene - GenBank accession number MF085557) with ~ 45 Kb, the only plasmid in this isolate. This isolate was obtained in Ribeirão Preto city, São Paulo state, from a soil sample cultivated with corn and soy, which are widely used for animal feeding in the Southeast region of Brazil.

Among the antibiotics recommended for *S. maltophilia* according to CLSI (2015), the isolate S431 was resistant to minocycline and sensitive for levofloxacin and trimethoprim-sulfamethoxazole by the disc diffusion method. According to the MIC tests, S431 was resistant to ceftazidime, presenting MIC > 256 μg/mL.

*S. maltophilia* is an opportunistic pathogen which presents a high level of with intrinsic resistance to different classes of antibiotics. It adapts easily to different environments, and has the ability to form biofilm. This species is most commonly associated with respiratory infection, but there are reports of different types of infections resulting in high mortality rates (Brooke 2012).

The *bla*_NDM-1_ gene was previously reported in a clinical isolate of *S. maltophilia* from China (Yang et al. 2014). The first report of NDM-producing bacterium was in 2009 in a clinical isolate from India (Yong et al. 2009). In Brazil, the first report was in 2013 in a *Providencia rettgeri* isolate and afterwards, other reports were described in enterobacterial species and also in NFGNB (Carvalho-Assef et al. 2013, Sampaio and Gales 2016). In the environment, NDM-producing bacteria have been reported, but there is only one report in the world of bacteria isolated from soil (Wang and Sun 2015). In isolates from different water sources, such as rivers, beach, recreational waters, wastewater and sewage, some reports in different countries have already occurred, including Brazil (Pagano et al. 2015, Kittinger et al. 2016, Islam et al. 2017, Mahon et al. 2017).

The *bla*_NDM_ gene has been detected in different plasmids, however IncA/C-type is one of the most prevalent. These plasmids stand out due to the ability of replication in different hosts and because they are commonly reported in multi-drug resistant (MDR) bacteria (Carattoli 2013). In Brazil, the association between the *bla*_NDM_ gene and IncA/C (Pereira et al. 2015) and other groups of plasmids such as IncH12, IncP, IncW, IncN and IncF has already been reported (Carvalho-Assef et al. 2014, Quiles et al. 2015).

The *bla*_NDM_ gene is increasingly being reported worldwide, predominantly in clinical isolates, where most of the studies are concentrated. There are few reports of *bla*_NDM_ in environmental bacteria, probably due to few studies in these sources, being the most of them focused on isolates from water (Walsh et al. 2011, Kittinger et al. 2016, Islam et al. 2017, Mahon et al. 2017). Among the β-lactam antibiotics, the carbapenems have a broad spectrum of action and are often used against infections caused by bacteria producing other β-lactamases (Meletis 2016). Resistance to these antibiotics by the production of β-lactamases, especially NDM, has become a major challenge in the treatment of different bacterial infections (Walsh and Toleman 2011).

The *bla*_NDM_ gene has been found in different plasmids, which frequently carries other resistance genes causing a rapid spread of these genes (Walsh et al. 2011, Berrazeg et al. 2014). As the *bla*_NDM-1_ gene was found inserted into a plasmid of a soil bacterium, it can potentially be transferred to other bacteria and disseminated in the environment and other sources, including water and humans, being for this reason a human health hazard. Besides that, due to the intrinsic resistance to imipenem, which it is associated with the MBL L1, *S. maltophila* can act as a silent disseminator of *bla*_NDM-1_, avoiding the detection of this gene in laboratories.

MDR bacteria are becoming widespread in the environment due to the incidence of antibiotic used in agriculture and these bacteria can migrate to the hospital environment. Since S431 was isolated from a soil sample cultivated with corn and soy, which are widely used for animal feeding in the Southeast region of Brazil, the *bla*_NDM-1_ can spread to different environments, which is of great concern. All reports in Brazil were in clinical isolates, with the exception of *Klebsiella pneumoniae* isolated from superficial beach water in Rio de Janeiro (Pagano et al. 2015).

To the best of our knowledge, there is only one study reporting NDM-producing *Acinetobacter calcoaceticus* and *Acinetobacter junii* in livestock soil samples from China (Wang and Sun 2015). As far as we know, this is the second report in the world and the first in Brazil of NDM-producing bacterium isolated from soil.
